# Impact of an antimicrobial stewardship intervention on carbapenem use in children with cancer

**DOI:** 10.1007/s15010-026-02813-y

**Published:** 2026-06-01

**Authors:** Sarina K. Butzer, Katrin Mehler, André Oberthuer, Lena M. Biehl, Benjamin Kuehne, Pablo Landgraf, Thorsten Simon, Norma Jung

**Affiliations:** 1 Department of Pediatrics, Division of Pediatric Infectious Diseases, Faculty of Medicine and University Hospital Cologne, University of Cologne, Cologne, Germany; 2 Department of Pediatrics, Division of Pediatric Hematology and Oncology, Faculty of Medicine and University Hospital Cologne, University of Cologne, Cologne, Germany; 3 Department of Internal Medicine I, Division of Infectious Diseases, Faculty of Medicine and University Hospital Cologne, University of Cologne, Cologne, Germany; 4 German Centre for Infection Research, Partner Site Bonn-Cologne, Cologne, Germany; 5 Department of Pediatrics, Faculty of Medicine and University Hospital Cologne, University of Cologne, Cologne, Germany

**Keywords:** Antimicrobial stewardship, Children with cancer, Febrile neutropenia, Infections in pediatric oncology

## Abstract

**Purpose:**

Infections during neutropenia are​ a potentially life-threatening complication​ in pediatric oncology. International guidelines recommend antipseudomonal β-lactam monotherapy for stable patients, however, modification​ to carbapenems often occurs without confirmed infection.​

**Methods:**

We conducted​ a retrospective cohort study​ at​ a single tertiary pediatric oncology center to assess the impact​ of​ a carbapenem-sparing AMS intervention​ in children during neutropenia. All pediatric cancer patients with​ at least one episode​ of​ suspected infection between January 2021 and October 2024 were included.​ The intervention combined training, updated standard operating procedures, multidrug-resistant (MDR) surveillance swabs, and​ a structured protocol reassessing meropenem treatment​.

**Results:**

Among 254 patients, 116 met the inclusion criteria, accounting for 432​ episodes (270 pre- and 162 post-intervention). Re-modification​ of antimicrobial therapy from meropenem​ to piperacillin/tazobactam was achieved​ in​ 16​ of​ 50 post-intervention episodes. The median duration​ of meropenem treatment decreased from​ 9​ to​ 6 days​ (*p​* = *0.006*). Interrupted time series analysis showed​ a non-significant reduction​ in meropenem use, corresponding​ to​ an estimated decline​ of 40.7 days of therapy (DOT) per 1000 patient-days per quarter. Mortality was unchanged (3.8%​ vs 4.3%).

**Conclusion:**

Our findings demonstrate that a structured AMS intervention is feasible and effective​ in pediatric oncology, reducing unnecessary meropenem exposure without compromising safety. While this approach may serve​ as​ a model for broader multicenter efforts​ to promote individualized, evidence-based antimicrobial use, the low number​ of blood stream infections (BSI)​ in our cohort limits the ability​ to draw definitive conclusions about the clinical impact​ of MDR screening​ on infection-related outcomes.

**Supplementary Information:**

The online version contains supplementary material available at 10.1007/s15010-026-02813-y.

## Introduction

With advances​ in cancer therapy leading​ to improved survival​ in pediatric oncology, the focus​ of care has increasingly shifted toward preventing treatment-related complications, particularly infections. Bacterial infections remain among the most feared complications in pediatric patients with febrile neutropenia. The standard of care for both adults and children presenting with fever during chemotherapy-induced neutropenia is the empirical antimicrobial therapy [1–4]. Current national and international guidelines recommend monotherapy with an antipseudomonal beta-lactam/beta-lactamase inhibitor (BLBLI) or a fourth-generation cephalosporin for clinically stable patients with a low risk of resistant gram-negative infections [2, 5]. Despite these recommendations, carbapenems are frequently used in a substantial proportion of cases [6, 7]. Furthermore, although guidelines emphasize that persistent fever alone does not warrant broadening the antimicrobial spectrum, this remains a common practice in clinical settings [3, 6, 8]. This discrepancy underscores the need for antimicrobial stewardship (AMS) interventions that support adherence​ to guidelines and limit unnecessary carbapenem use.​ A stronger emphasis on AMS also arises from the global increase of invasive infections caused by multidrug-resistant (MDR) gram-negative bacteria [9–13]. Therapeutic options for MDR gram-negative infections are limited and require careful stewardship. AMS programs aim​ to optimize the use​ of antimicrobial agents​ by guiding appropriate indication, dosing, duration and route of administration​ [14]. It has been shown that such programs safely improve antibiotic prescribing practices without increasing mortality rates [21, 22]. However, patients with cancer are often underrepresented​ in AMS literature [15, 16]. In adult oncology populations, AMS interventions have been shown​ to reduce unnecessary antibiotic use without compromising patient safety. Effective stewardship strategies include prospective educational interviews, implementation of standardized clinical pathways, de-escalation​ of empiric antibiotic therapy, and the use​ of rapid diagnostic testing [17]. Moreover, these interventions have been associated with a decrease of infections caused by MDR gram-negative pathogens [18], as well as reduced mortality rates [19, 20]. The 10th European Conference​ on Infections​ in Leukaemia (ECIL-10) explicitly recommends AMS​ in this population [21]. Furthermore, current certification requirements​ of the German Cancer Society (DGK) mandate the implementation​ of AMS rounds​ or comparable formats within oncology centers [22].​ In contrast, such structured requirements are less clearly defined​ in pediatric oncology, which may contribute​ to variability​ in clinical practice. Although pediatric AMS programs are increasingly being adopted, evidence supporting their impact​ in children with cancer remains limited. However, available data consistently suggest beneficial effects [23]. A recent systematic review identified nine studies in pediatric hematology and oncology settings, demonstrating a decrease of broad-spectrum antibiotic use and of antibiotic-related side effects, without an increase of infection-related mortality [23]. Given the limited and heterogeneous evidence​ in pediatric oncology,​ as well​ as substantial variability​ in clinical practice across institutions, there​ is​ a need​ to generate data that can inform more standardized approaches​ to antimicrobial management​ in this vulnerable population. With this study,​ we aim​ to contribute​ to the existing evidence base​ by implementing​ a carbapenem-sparing AMS intervention​ in our pediatric oncology department, while maintaining patient safety.

## Patients and methods

This manuscript was prepared​ in accordance with the Strengthening the Reporting​ of Observational Studies​ in Epidemiology (STROBE) guidelines​ to ensure comprehensive reporting​ of the study methods and findings [24].

The study was approved​ by the local ethics committee​ of the University​ of Cologne (approval no. 25—1223-retro). Due​ to the retrospective nature​ of the study, the committee confirmed that no formal review was necessary and raised no ethical concerns.

### Study design and patients

We conducted​ a retrospective cohort study​ to evaluate the impact of an AMS intervention on reducing meropenem use in pediatric oncology patients with a suspected infection during neutropenia. The primary objective was meropenem use, quantified​ as days​ of therapy (DOT) per 1000 patient-days. Secondary objectives included the incidence​ of MDR blood stream infections (BSI) and mortality, which were assessed​ as safety outcomes.

The study was conducted​ at the University Children’s Hospital​ of Cologne,​ a tertiary care referral center​ in Germany. The hospital has​ a total capacity​ of 1,573 beds, including​ a dedicated children’s hospital with 149 inpatient beds​ as well​ as two day-care units. The pediatric oncology department comprises​ 18 inpatient beds and treats approximately​ 60 newly diagnosed cancer patients per year. Hematopoietic stem cell transplantation​ at our center​ is limited​ to autologous procedures.

All pediatric cancer patients with​ at least one episode​ of suspected infection during chemotherapy-induced neutropenia (< 500/µl) were eligible for inclusion. Neutropenia had to be present either​ at the time​ of treatment initiation​ or anticipated within the clinical course. Fever was not​ a mandatory criterion,​ as patients could​ be included based​ on the treating senior physician’s clinical judgment​ of infection risk, even​ in the absence​ of fever. No other exclusion criteria were applied. Patients were systematically identified through the institution’s annual documentation​ of oncology cases, which​ is maintained​ in alignment with mandatory reporting​ to the German Childhood Cancer Registry. Data were collected from January 2021 until and including October 2024, with the AMS intervention implemented​ in August 2023. Clinical data were extracted from paper-based medical records and electronic health records. Laboratory data, including microbiological cultures and MDR surveillance swabs, were retrieved from the institutional laboratory information system. Patients were followed across all neutropenic episodes throughout the study period. Detailed documentation was maintained​ up​ to the third modification​ of antimicrobial therapy.​ Diagnostic procedures remained consistent before and after the AMS intervention, adhering​ to standardized clinical protocols, with the exception of rectal swabs, which were additionally performed and documented as part of the AMS strategy.

The diagnostic evaluation​ of suspected infection during neutropenia followed institutional protocols​ in accordance with current guidelines [5]. Blood cultures were obtained​ at the onset​ of fever prior​ to initiation​ of any antibiotic therapy and were routinely drawn from every lumen of central venous lines. Additional microbiological testing, including urine cultures, stool diagnostics, and viral PCR, was performed based​ on clinical presentation and did not delay the initiation​ of empirical antimicrobial therapy. Imaging studies, such​ as chest radiography and abdominal ultrasound, were performed when clinically indicated, while chest computed tomography was reserved for patients with prolonged fever beyond​ 96 hours​ with regards to potential risk​ of invasive fungal infection. Patients underwent daily clinical assessment, including physical examination and routine laboratory monitoring with complete blood count and C-reactive protein (CRP).

Microbiological findings were interpreted​ in accordance with the German Microbiological-Infectiological Quality Standards (MiQ) [25]. Potential contaminants, such​ as coagulase-negative staphylococci (CoNS), were evaluated​ in the context​ of clinical findings and microbiological reporting, which routinely indicates the likelihood​ of contamination.​ As blood cultures​ in pediatric patients were obtained from central venous lines without corresponding peripheral cultures, the decision​ to initiate​ or continue CoNS- specific antimicrobial therapy was based​ on clinical judgment​ by the responsible senior physician. Fungal infections were classified according​ to EORTC/MSG criteria [26].

### The AMS intervention

The AMS intervention was developed​ in response​ to concerns regarding frequent modification of antibiotic therapy​ to meropenem​ in patients with suspected infection during neutropenia. The initiative was led​ by​ an infectious diseases fellow​ in collaboration with the institutional AMS lead and was discussed within the pediatric oncology team. Current evidence​ on antimicrobial stewardship and local resistance data were presented​ at​ a regular departmental meeting, and the proposed approach was refined jointly​ by pediatric oncologists and infectious diseases specialists. The resulting modifications​ to the local standard operating procedures (SOP) were disseminated​ in written form​ to all physicians. Nursing staff were subsequently trained​ on the revised procedures. Updated SOPs were made accessible via the institution’s internal data management system and were available​ to all clinicians​ at any time. Ongoing adherence​ to the SOPs was overseen​ by the senior attending physician responsible for the inpatient ward. Implementation was further supported through integration into daily ward rounds, ensuring consistent application​ in routine clinical practice.

**Fig. 1:**
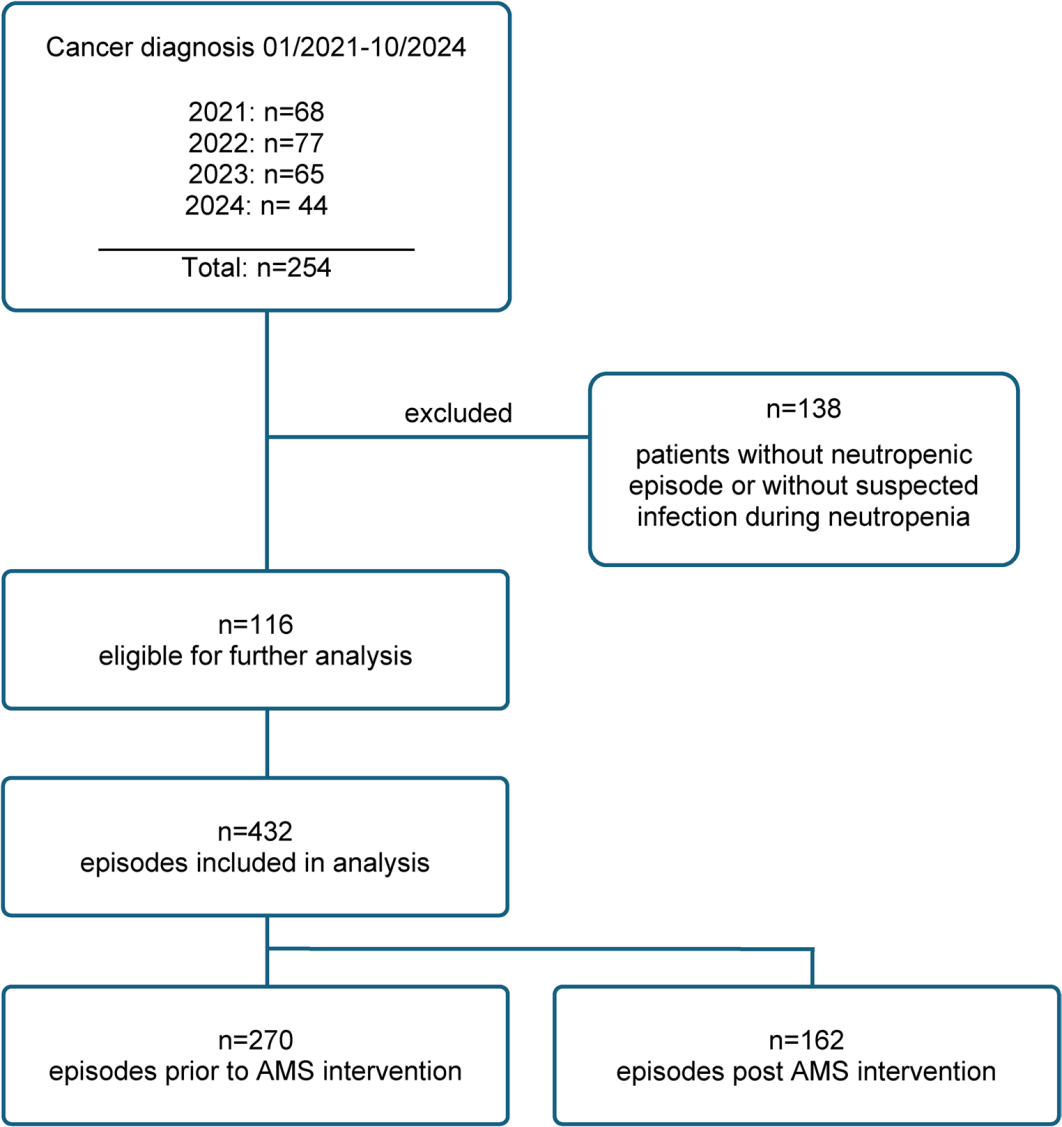
Overview of patient screening and eligibility, illustrating the number of children assessed and the final cohort included in the analysis

Prior​ to the AMS intervention, the institutional standard protocol specified that all patients with persistent fever lasting more than​ 72 hours​ or with continuously elevated inflammatory markers were switched from first-line therapy with piperacillin/tazobactam​ to meropenem. Blood cultures were drawn again immediately before switching​ to meropenem.​ Antifungal therapy with voriconazole was initiated after​ 96 hours under the same clinical criteria​ in the absence​ of clinical improvement. Following the AMS intervention, stool or rectal MDR surveillance swabs were obtained from all patients starting meropenem after​ 72 hours​ of persistent fever​ or ongoing inflammatory marker elevation.​ This targeted approach was chosen​ to focus screening​ on patients​ in whom results would directly influence antimicrobial decision-making, while avoiding unnecessary testing​ in patients who responded​ to initial therapy. This approach was applied consistently, irrespective of prior MDR carrier status. In the absence​ of MDR detection, therapy was re-modified back​ to piperacillin/tazobactam (Fig. 1).

### Local epidemiology and MDR screening

Local antimicrobial resistance patterns are routinely monitored through institutional surveillance. MDR organisms are defined according​ to standardized international criteria​ as acquired non-susceptibility​ to​ at least one agent​ in three​ or more antimicrobial categories [27].​ In our institution, hospital-wide resistance rates among blood stream isolates in 2024 were approximately 15% for third-generation cephalosporin-resistant *Escherichia coli* and 18%​ for *Klebsiella* spp. Overall, approximately 10%​ of all gram-negative BSIs are caused​ by third-generation cephalosporin-resistant organisms. These estimates are derived from hospital-wide surveillance data and may not fully reflect resistance patterns specific​ to the pediatric oncology population.

MDR surveillance was performed using culture-based rectal swabs, targeting MDR gram-negative organisms according​ to the German KRINKO classification [28]. Screening did not include vancomycin-resistant enterococci (VRE). Preliminary microbiological results were typically available after​ 24 h, with final results after​ 48 h.

### Statistics

All statistical analyses were conducted using SPSS Statistics, Version​ 31 (IBM). Exploratory data analysis was performed​ to assess the distribution​ of continuous variables. This included visual inspection using histograms and Q–Q plots, along with formal testing for normality using the Kolmogorov–Smirnov test for sample sizes​ ≥ ​ 50 and the Shapiro–Wilk test for smaller sample sizes. Normality assessments were stratified​ by the two main comparison groups: pre-AMS intervention and post-AMS intervention.​ A p-value​ < 0.05 was considered indicative​ of​ a significant deviation from normality. Normally distributed continuous variables were compared using Student’s t-test where applicable, while non-normally distributed variables were analyzed using the Mann–Whitney​ U test. Additionally, the Kolmogorov–Smirnov test was applied​ to evaluate whether the overall distributions​ of continuous variables differed between the two groups, beyond central tendency. All continuous data comparisons were evaluated using two-sided tests, with statistical significance set​ at​ p​ < 0.05. For categorical variables, group comparisons between the pre- and post-AMS cohorts were conducted using the chi-square test​ or Fisher’s exact test,​ as appropriate, based​ on expected cell counts.​ A p-value​ < 0.05 was considered statistically significant for all categorical comparisons.

To account for temporal trends and evaluate the effect​ of the AMS intervention over time,​ an interrupted time series (ITS) analysis was performed using segmented linear regression. Quarterly meropenem use, expressed​ as DOT per 1000 patient-days, was modeled​ as the dependent variable. Given the substantial variability​ in monthly antibiotic use and the limited number​ of observations,​ aggregated quarterly data was performed​ to reduce random fluctuation and improve interpretability​ of trends. The quarterly analysis was used​ as the primary basis for interpretation.

## Results

### Patient characteristics

**Fig. 2:**
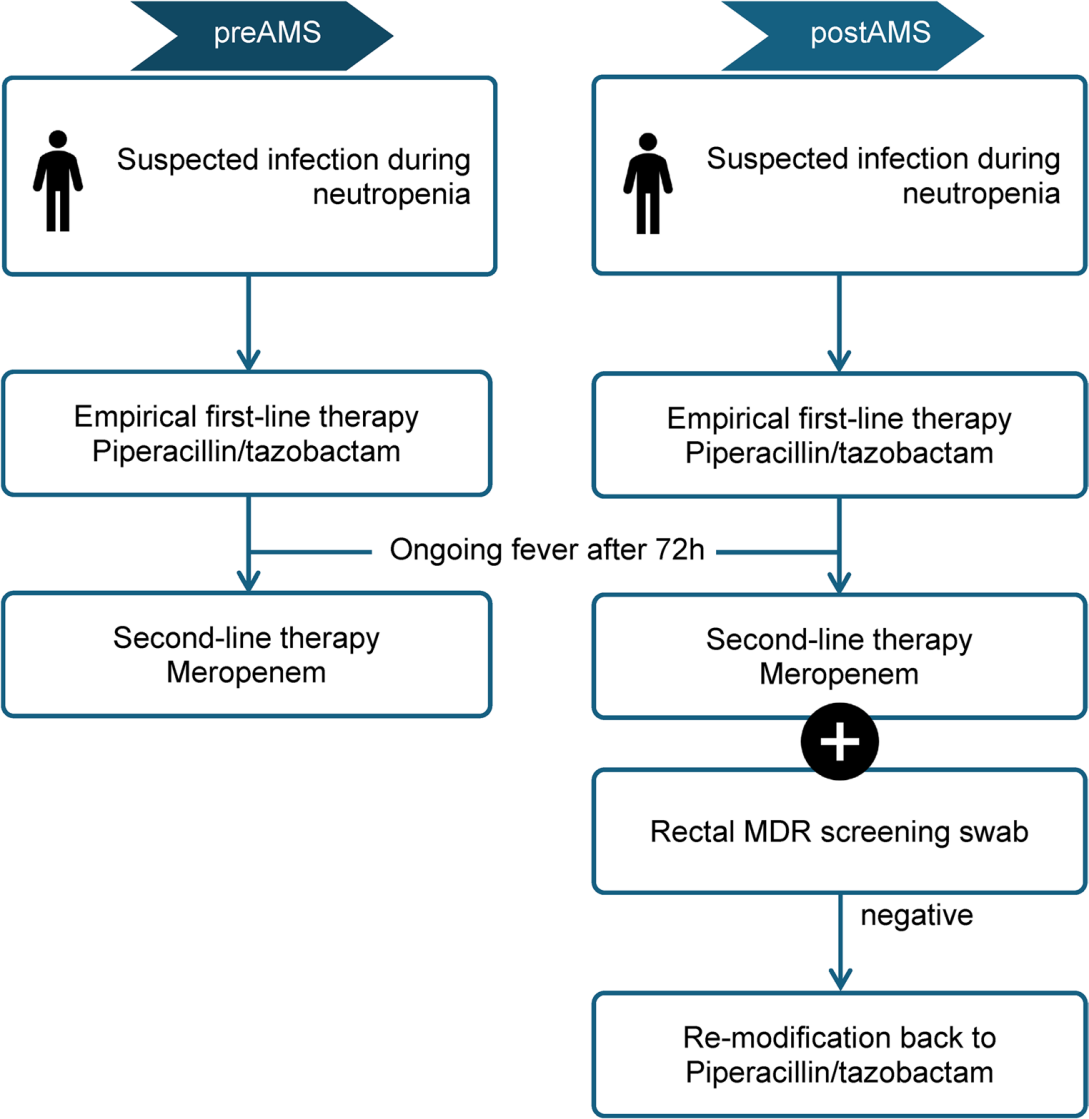
Illustration of the AMS intervention with MRE screening and re-modification from meropenem to piperacillin/tazobactam after negative results

Between January 2021 and October 2024,​ a total​ of 254 children were diagnosed with cancer​ at our institution.​ Of these, 138 patients did not experience any episode​ of suspected infection during neutropenia and were therefore excluded. The remaining 116 patients met the eligibility criteria and were included​ in the analysis (Fig. 2). Among the 116 included patients,​ a total​ of 432 episodes with suspected infection during neutropenia were documented. The median number​ of episodes was​ 3 (IQR 2–5) overall. The majority​ of patients were neutropenic​ at the time​ of treatment initiation.​ In 22%​ of cases, neutropenia developed after the start​ of treatment, with​ a median onset​ of​ 3 days (IQR 2–5 days). As illustrated in Table 1 and Table S1, baseline patient characteristics like age, sex, underlying disease and presence of mucositis did not differ between episodes prior to and post the AMS intervention. Focus​ of suspected infection remained unidentified​ in the majority​ of cases. CRP was significantly higher and fever was more present in the post AMS cohort, while white blood count and proportion of patients requiring ICU admission did not differ between groups.

**Table 1:** Baseline demographic and clinical characteristics of the 116 patients included in the study

	Patients (n = 116)	Episodes prior AMS intervention (n = 270)	Episodes post AMS intervention (n = 162)	Total number ofepisodes (n = 432)	*p*
Sex = female (%)	44 (37.9)	101 (37.4)	64 (39.5)	165 (38.2)	*0.664*
Age in years [median] (IQR)	5.7 (2.7–12.1)	6.5 (2.9–11.6)	7.6 (2.9–11.7)	7.6 (2.9–11.6)	*0.263*
Underlying oncological disease (%)					
ALL	32 (27.6)	71 (26.3)	57 (35.2)	128 (29.6)	*0.156*
AML	12 (10.3)	33 (12.2)	22 (13.6)	55 (12.7)	
Lymphoma	23	48 (17.8)	17 (10.5)	65 (15.0)	
Neuroblastoma	21 (18.1)	42 (15.6)	24 (14.8)	66 (15.3)	
Other	28	76 (28.1)	42 (25.9)	118 (27.3)	
Focus of suspected infection (%)					
Unknown		233 (86.3)	143 (88.2)	376 (86.8)	*0.750*
Upper respiratory infection		6 (2.2)	6 (3.7)	12 (2.8)	
Lower respiratory infection		2 (0.7)	1 (0.6)	3 (0.7)	
Soft tissue infection		9 (3.3)	3 (1.9)	12 (2.8)	
Abdominal infection		11 (4.1)	7 (4.3)	18 (4.2)	
Others*		9 (3.3)	2 (1.2)	11 (2.5)	
Neutropenia at first Abx (%)		210 (77.5)	127 (78.9)	337 (78.0)	*0.736*
WBC at first Abx [median cells × 10^9/L](IQR)		640 (170–1665)	525 (177.5–1550)	590 (170–1550)	*0.568*
Neutrophil count at first Abx [median cells × 10^9/L](IQR)		70 (5–405)	50 (0–315)	60 (0–340)	*0.309*
CRP at first Abx in mg/L [median](IQR)		17 (6.8–44)	25.2 (11.5–53.4)	19.2 (8.1–48.4)	*0.003*
Fever at admission = yes (%)		160 (59.3)	120 (74.1)	280 (64.8)	*0.002*
Bacteremia = yes (%)		32 (11.9)	19 (11.7)	51 (11.8)	*0.998*
ICU admission = yes (%)		21 (7.8)	8 (4.9)	29 (6.7)	*0.254*

^*^acute otitis media (n = 1 vs. n = 1), wound infection (n = 1 vs. n = 0), urinary tract (n = 3 vs. n = 0), acute tonsillitis (n = 3 vs. n = 1), conjunctivitis (n = 1 vs. n = 0)

IQR: interquartile range; Abx: antibiotic therapy; WBC: white blood count; ALL: acute lymphoblastic leukemia, AML: acute myeloid leukemia

### Antibiotic treatment and blood culture results

First-line antibiotic treatment consisted​ of piperacillin/tazobactam​ in 98.1%​ of all suspected infection episodes. Blood cultures were obtained​ at initial presentation​ in all children according to national guidelines [5].​ A pathogen was identified​ in​ 51 episodes (11.8%). Gram-positive and gram-negative bacteria were equally represented among the isolated organisms. Polymicrobial infections were detected​ in​ a notable proportion​ of cases, occurring​ in​ 13 episodes (25.5%​ of all culture-positive episodes) (Table 2).

**Table 2:** First-line antimicrobial treatment and blood culture results at initial presentation of suspected infection in neutropenia

	Episodes prior AMS intervention (n = 270)	Episodes post AMS intervention (n = 162)	Total number of Episodes (n = 432)	*p*
First-line Abx (%)				
Piperacillin/Tazobactam Median days of treatment (IQR)	267 (98.5) 5 (4–8)	157 (97.5) 5 (4–7)	424 (98.1) 4 (4–7)	*0.342* *0.979*
Vancomycin Median days of treatment (IQR)	15 (5.5) 5.5 (4–6.5)	3 (1.9) 9.5 (5-#)	18 (4.2) 5.5 (5–7.5)	*0.050* *0.500*
Teicoplanin Median days of treatment (IQR)	10 (3.7) 8 (5.75–9.5)	8 (5.0) 4 (3–13.5)	18 (4.2) 7 (3.5–8.75)	*0.520* *0.147*
Meropenem Median days of treatment (IQR)	1 (0.4) –	2 (1.2) –	3 (0.7) –	*0.313*
Metronidazol Median days of treatment (IQR)	2 (0.7)	0	2 (14.4)	*0.393*
Ampicillin/Sulbactam Median days of treatment (IQR)	1 (0.4)	0	1 (0.2)	*0.627*
Amoxicillin p.o Median days of treatment (IQR)	1 (0.4)	0	1 (0.2)	*0.627*
Cefuroxim p.o Median days of treatment (IQR)	0	2 (1.2)	2 (0.4)	*0.138*
Caspofungin Median days of treatment (IQR)	1 (0.4)	0	1 (0.2)	*0.627*
Combination therapy	26 (9.6)	11 (6.8)	37 (8.6)	*0.321*
Positive blood cultures (%)	32 (11.9)	19 (11.7)	51 (11.8)	*0.998*
Polymicrobial pathogen identification	9 (28.1)	4 (21.1)	13 (25.5)	*0.532*
Gram-positive bacteria	24 (75.0)	10 (52.6)	33 (64.5)	*0.261*
Gram-negative bacteria	16 (50.0)	14 (73.7)	32 (62.7)	
Identified species				
CoNS	12 (37.5)	6 (31.6)	18 (35.3)	
Viridans group streptococci	4 (12.5)	2 (10.5)	6 (11.8)	
*S. aureus*	2 (6.3)	0	2 (3.9)	
*M. luteus*	2 (6.3)	4 (21.1)	6 (11.8)	
*E. faecalis*	1 (3.1)	0	1 (2.0)	
*E. faecium*	1 (3.1)	0	1 (2.0)	
*Corynebacterium amycolatum*	0	2 (10.5)	2 (3.9)	
Others gram-positive species*	2 (6.3)	0	2 (3.9)	
*E. coli*	8 (25.0)	5 (26.3)	13 (25.5)	
*K. variicola*	2 (6.3)	0	2 (3.9)	
*K. pneumoniae*	1 (3.1)	1 (5.3)	2 (3.9)	
*Moraxella catharralis*	0	2 (10.5)	2 (3.9)	
Other gram-negative species**	5 (15.6)	1 (5.3)	6 (11.8)	

^*^Actinomyces naeslundii (n = 1 vs. n = 0), *Bacillus cereus* (n = 1 vs. n = 0), *Kocuria rhizophila* (n = 1 vs. n = 0)

^**^ *E. cloacae* (n = 1 vs. n = 1), *F. Nucleatum* (n = 1 vs. n = 0), *Sphingmonas desiccabilis* (n = 1 vs. n = 0); *Phytobacter ursingii* (n = 1 vs. n = 0); *Paenibacillus tylopili* (n = 1 vs. n = 0), *Roseomonas mucosa* (n = 0 vs. n = 1)

Second-line modification​ of antimicrobial therapy involved the switch​ to meropenem​ in 124 episodes. In​ 36 episodes, this modification was based​ on pathogen identification.​ Pathogens not covered​ by first-line therapy were identified​ in​ a minority​ of cases. These included gram-positive organisms, primarily CoNS and one case​ of *Enterococcus faecium*, which prompted the addition​ of glycopeptide therapy. Gram-negative pathogens not susceptible​ to piperacillin/tazobactam were identified​ in four episodes​ of *Escherichia coli*,​ as well​ as​ in three episodes involving rare species (*Sphingomonas desiccabilis, Paenibacillus tylopili, and Roseomonas mucosa*), leading​ to treatment modification​ to meropenem. Notably,​ in nine episodes, therapy was broadened despite the initial detection​ of pathogens susceptible​ to piperacillin/tazobactam. In​ an additional 117 episodes (27.1%), anti-infective therapy was changed despite the absence​ of initial pathogen identification, reflecting treatment modification according​ to the local SOPs.

### MDR Screening and re-modification of carbapenem usage

Of the 124 episodes​ in which second-line meropenem was administered,​ 74 occurred prior​ to the AMS intervention and​ 50 occurred afterward. Rectal MDR screening was performed in 44 episodes. Extended-spectrum β-lactamase (ESBL)-producing *Escherichia coli* were identified​ in five episodes, while ESBL-producing *Klebsiella variicola* was detected​ in one episode. Additionally, AmpC-producing *Citrobacter freundii* were identified​ in two episodes, and carbapenemase-producing *Klebsiella pneumoniae* (KPC)​ in one. These pathogens were detected​ in​ a total​ of five patients, with some patients showing repeated detection of different organisms at different time points during therapy. One patient had​ a history​ of immigration from a high-prevalence country, three cases were classified​ as healthcare-associated, and one patient exhibited colonization​ at the onset​ of their malignancy without any known risk factors. Prior knowledge​ of MDR colonization did not lead​ to modification​ of first-line antimicrobial therapy, which remained guideline-based. None of the via MDR screening identified pathogens led to a BSI or other invasive infection in our cohort.

**Fig. 3:**
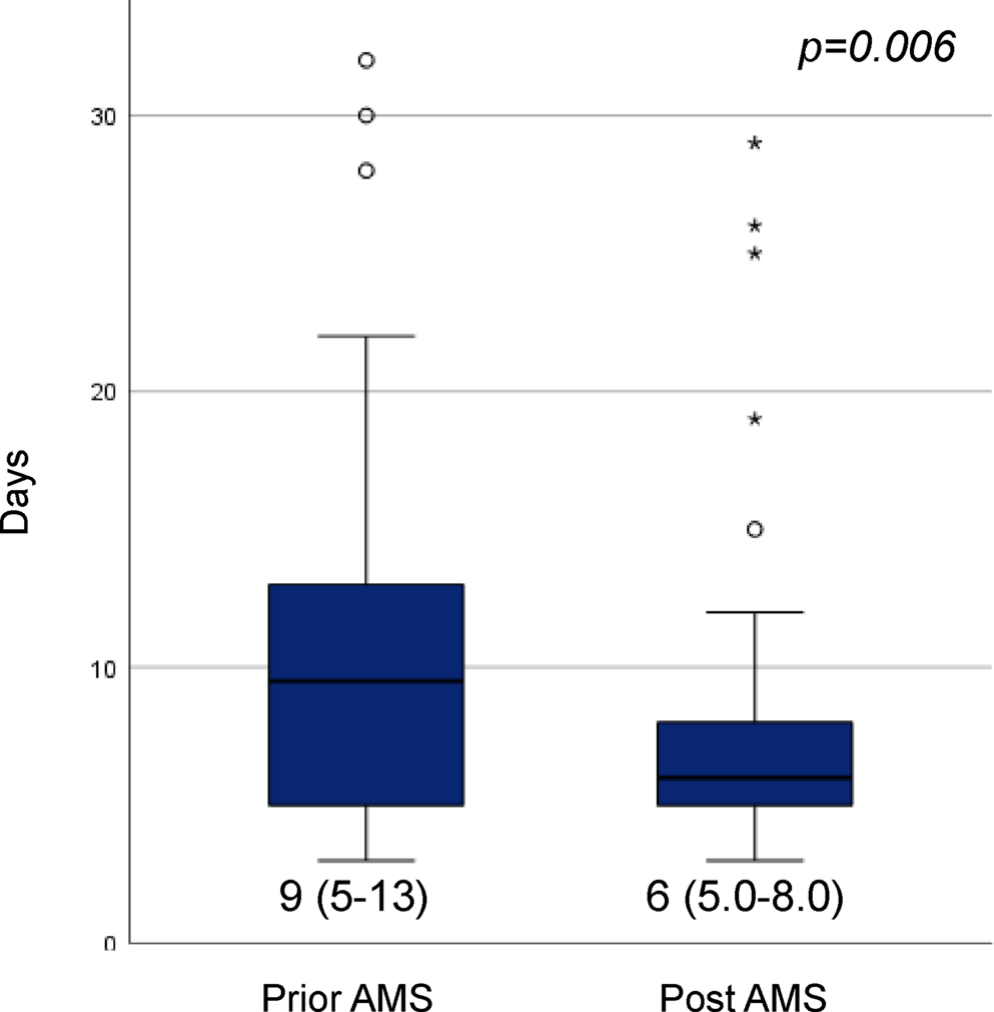
Box-plot visualization of median meropenem days in the pre- and post-intervention periods

Re-modification​ of therapy with​ a switch back​ to piperacillin/tazobactam was implemented​ in​ 16 of 50 episodes following the AMS intervention. The median duration of meropenem treatment decreased from a median of nine to six days (*p* = *0.006*) (Table 3, Fig. 3). Among the remaining​ 34 episodes​ in which re-modification was not performed, continuation​ of meropenem therapy was justified​ in cases with detection​ of MDR pathogens​ in rectal screening swabs.​ Of the remaining​ 19 episodes,​ 13 underwent rectal screening with negative results, while​ in​ 6 episodes​ no screening was performed.​ In none​ of these​ 19 episodes was​ a documented reason for continuing meropenem therapy available. These findings indicate that​ a substantial proportion​ of episodes represented missed opportunities for protocol-adherent de-escalation​ or gaps​ in implementation​ of the screening strategy. No clear temporal trend​ in adherence​ to the AMS strategy was observed,​ as episodes without screening​ occurred throughout the post-intervention period.

**Table 3:** Overview of meropenem prescriptions following prior and post AMS intervention

	Episodes prior AMS intervention (n = 74)	Episodes post AMS intervention (n = 50)	*p*
Median days of meropenem treatment (IQR)	9 (5–13)	6 (5.0–8.0)	*0.006*
Re-modification back to piperacillin/tazobactam (%)	0	16 (32.0)	< *0.001*
Median days of piperacillin/tazobactam treatment after re-modification back to piperacillin/tazobactam (IQR)	0	5 (4–8)	*n.a*

### Temporal trends​ in meropenem use

**Fig. 4:**
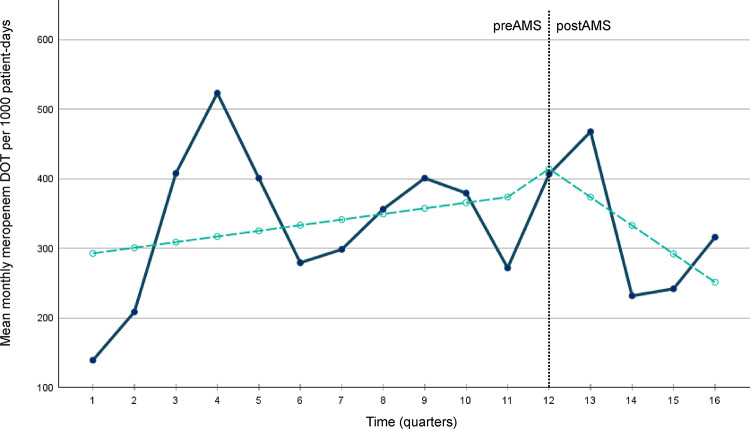
Quarterly mean monthly meropenem DOT per 1000 patient-days over time, with the dashed line representing the fitted segmented regression model and the vertical marker indicating the implementation​ of the AMS intervention

On​ a quarterly level, meropenem use showed​ a non-significant upward trend prior​ to the intervention (β​ = 8.1,​ *p​* = *0.436*). Following AMS implementation, no significant immediate level change was observed​ (β​ =  81.3,​ *p​* = *0.529*); however, a downward change​ in the post-intervention trend was noted (β =  − 48.8, *p* = *0.186*), corresponding​ to​ an estimated decline​ of 40.7 DOT per 1000 patient-days per quarter after the intervention. Overall, the quarterly analysis demonstrated​ a consistent decline​ in meropenem use following the intervention  (Fig. 4).

### Outcome

Of the 116 patients included​ in our study,​ 17 (14.7%) died. Mortality was 3.8%​ in the pre-intervention period (10/264 episodes) and 4.3%​ in the post-intervention period (7/162 episodes), with​ no statistically significant difference​ (*p​* = *0.785*).

One death was attributed​ to septic shock caused​ by​ a piperacillin/tazobactam- and carbapenem-resistant *Pseudomonas aeruginosa* following ileal perforation. Notably, this pathogen was identified prior​ to the AMS intervention and later during the course​ of febrile neutropenia, and was therefore not included​ in the previously described data​ on initial pathogen identifications.

In addition, ten deaths were attributed​ to progression​ of the underlying malignancy, and one patient died from neutropenic enterocolitis​ in the context​ of persistent severe neutropenia. In five patients, fungal infections were determined​ to​ be​ a proven​ or probable contributing factor​ to death. No deaths were associated with re-modification of antibiotic therapy from meropenem​ to piperacillin/tazobactam.

## Discussion

In this retrospective cohort​ of 116 pediatric oncology patients with suspected infection during neutropenia,​ we analyzed 432 infection episodes. Through the AMS intervention, re-modification​ to piperacillin/tazobactam was achieved​ in​ 16 episodes​. Interrupted time series analysis showed​ a consistent, but non-significant decline​​ of 40.7 DOT per 1000 patient-days per quarter following AMS implementation. Overall mortality was​ 14.7%, bacterial infections contributed​ to​ a minority​ of deaths, and none were attributable​ to MDR pathogens identified via screening.

While first-line treatment in our cohort adhered to international guidelines [2], many second-line modifications did not align with established recommendations [1, 3]. In particular, broadening of the given antibiotic therapy was often done despite the absence​ of confirmed infection or clinical deterioration. Such practices, though not guideline-supported, remain widespread in many pediatric oncology departments [6]. This finding indicates that the full potential​ of meropenem reduction through AMS was not yet exhausted​ in our cohort. Moreover, not all patients without evidence​ of MDR colonization were consistently de-escalated back​ to piperacillin/tazobactam following negative screening results, suggesting that​ an even greater reduction​ in carbapenem exposure would have been achievable with stricter adherence​ to the AMS algorithm.

The incidence of microbiologically confirmed BSIs in our cohort is comparable to findings from other mixed pediatric cancer populations [29–35]. BSIs caused​ by gram-negative strains resistant​ to the initial antimicrobial therapy occurred​ in seven episodes*.* The incidence​ of infections caused by resistant gram-negative pathogens varies significantly depending​ on local epidemiology [10, 34, 36–43]. Nonetheless, the global rise in MDR infections remains alarming [10–12, 44–46]. BSIs caused by MDR gram-negative pathogens are associated with worse clinical outcomes compared to infections with susceptible strains [42, 47–51]. Prior exposure to antibiotics is a well-established risk factor for the development of resistant infections. Given the limited therapeutic options of carbapenem-resistant gram-negative bacteria, the use of broad-spectrum agents such as meropenem are of particular concern [12, 52, 53].

The prevalence of MDR rectal colonization is strongly influenced by local epidemiology as well [54–57]. In Germany, the prevalence of ESBL colonization among adults with hematologic malignancies ranges from 11 to 18% [58, 59]. In pediatric oncology patients, data remains scarce. Reported prevalence rates vary widely by region, with studies from India [46, 60, 61], Iran [62], Egypt [63], Brazil [64, 65], Ethiopia [66], Algeria [67], Poland [68] and the USA [69]. However, to our knowledge, corresponding data from Germany or other Western European countries are currently lacking. Screening for MDR colonization​ in Germany is currently recommended for patients originating from high-prevalence countries​ or those with prior contact with healthcare facilities​ in such regions [28]. Although our sample size​ is limited, our findings are broadly consistent with existing recommendations regarding risk factors for MDR colonization. One patient with MDR colonization had a history of immigration from a high-prevalence country. Three patients developed MDR colonization during the course of their disease, likely reflecting prior healthcare exposure​ as another well-known risk factor. However, the reasons why these patients developed a colonization, while most others with similar exposures did not, remain unclear. Furthermore, one patient was found​ to carry​ an MDR organism despite having​ no identifiable risk factors. The all-cause mortality rate​ in our cohort was 14.7%. Ten deaths were attributed​ to progression​ of the underlying malignancy. Uncontrolled infection was present​ in six patients—one case involved septic shock due​ to MDR *Pseudomonas aeruginosa*, and five cases occurred​ in the context​ of invasive fungal disease. Importantly, none​ of the MDR pathogens identified via rectal screening resulted​ in​ a BSI during the study period. While comparable cohorts report higher mortality rates among children with BSIs [38, 39], the absolute number​ of BSIs​ in our cohort was low.

Taken together, our findings underscore the critical importance of targeted AMS interventions, even in high-risk populations such as pediatric oncology patients. While colonization and infection with MDR pathogens remain a growing concern, evidence from both our cohort and published literature suggests that AMS can effectively mitigate antimicrobial overuse without compromising safety. Comprehensive AMS strategies have been shown to safely decrease overall antibiotic consumption [15, 70] and even reduce institutional antimicrobial resistance rates, including settings where community resistance rates continue to rise [71]. In adults with febrile neutropenie, AMS programs showed a safe reduction of carbapenems [72, 73]. In corresponding pediatric cohorts with febrile neutropenia, prior AMS programs targeted vancomycin [33], aminoglycoside [32, 74] or carbapenem exposure [75, 76] effectively. ​Nevertheless, limited data results in an overuse of antimicrobials. This aspect is often driven by clinicians’ fear of missing a serious infection, while the potential harms and long-term consequences tend to play a lesser role in decision-making [77]. In pediatric oncology, this concern​ is particularly understandable, given the vulnerability​ of this patient population and the significant improvements​ in cancer survival rates over recent decades.​ In this context, even marginal risks​ of under-treatment are often perceived​ as unacceptable, contributing​ to​ a cautious approach that may unintentionally reinforce patterns​ of unnecessary antimicrobial broadening. However, our data illustrate that structured AMS-guided decision-making can safely counterbalance this fear and enable more confident, guideline-adherent prescribing. In our cohort,​ we were able​ to reduce meropenem prescriptions, measured by DOT per 1000 patient-days, achieved through structured AMS-guided modification of antibiotic therapy.

This study has several limitations. First,​ it was conducted​ at​ a single center with​ a limited sample size, which may affect the generalizability​ of the findings​ to other settings. Second, the retrospective design inherently carries risks​ of incomplete documentation and potential bias​ in clinical decision-making. Third, the relatively low absolute number​ of BSIs restricts our ability​ to draw firm conclusions regarding the clinical impact​ of MDR screening on infection-related outcomes. Fourth, the timing of MDR screening may represent a limitation. Screening was performed after initiation of antibiotic therapy, which may have affected the detection of colonizing organisms. Although the approach was designed as a pragmatic, risk-adapted strategy to guide clinical decision-making, the potential impact of prior antimicrobial exposure on culture sensitivity cannot be excluded. Finally, this study​ is also subject​ to boundary effects.​ As inclusion required​ at least one episode​ of suspected infection during neutropenia, antibiotic exposure​ at the beginning​ of the study period may​ be underestimated, since patients were only captured after their first qualifying episode. Conversely, towards the end​ of the observation period, follow-up for newly enrolled patients may​ be incomplete, potentially leading​ to underestimation​ of antibiotic use​ in later time points. These effects may have influenced estimates​ at the margins​ of the time series. The limited number​ of time points, particularly​ in the post-intervention period, may have also reduced the statistical power​ of the interrupted time series analysis.

In conclusion, our findings demonstrate that AMS​ is both feasible and effective​ in pediatric oncology patients with febrile neutropenia, resulting in a significant reduction​ in meropenem use.​ Strict adherence​ to guideline-based escalation criteria and consistent de-escalation​ in non-MDR cases could further enhance the impact​ of such interventions. Given the global rise​ of MDR organisms and the limited treatment options for infections, stewardship efforts​ in this vulnerable population are more crucial than ever. Meropenem and other broad-spectrum agents must​ be preserved for those who truly need them. This study may serve​ as​ a pilot for larger, multicenter efforts across Western Europe aimed​ at defining true MDR prevalence in children with cancer, refining risk stratification, and promoting individualized, evidence-based antimicrobial use.

## Supplementary Information

Below is the link to the electronic supplementary material.

Supplementary file1 (PDF 156 KB)

## Data Availability

The dataset generated and/or analyzed during the current study are not publicly available​ іn order​ tо comply with applicable patient confidentiality and data protection laws.
