# Supplementary material for: Impact of an antimicrobial stewardship intervention on carbapenem use in children with cancer
Source: Infection. 2026 Jun 1;54(4):1931–43. doi: 10.1007/s15010-026-02813-y (PMC13476331; doi:10.1007/s15010-026-02813-y)
Supplement: Supplementary file 1 [file 15010_2026_2813_MOESM1_ESM.pdf]

# Impact of an Antimicrobial Stewardship Intervention on Carbapenem Use in Children with Cancer

Sarina K. Butzer[1,2], Katrin Mehler[1], André Oberthuer[1], Lena M. Biehl[3,4], Benjamin Kuehne[5], Pablo Landgraf[2], Thorsten Simon[2], Norma Jung[3]

1 University of Cologne, Faculty of Medicine and University Hospital Cologne, Department of Pediatrics, Division of Pediatric Infectious Diseases

2 University of Cologne, Faculty of Medicine and University Hospital Cologne, Department of Pediatrics, Division of Pediatric Hematology and Oncology

3 University of Cologne, Faculty of Medicine and University Hospital Cologne, Department of Internal Medicine I, Division of Infectious Diseases

4 German Centre for Infection Research, Partner Site Bonn-Cologne, Germany

5 University of Cologne, Faculty of Medicine and University Hospital Cologne, Department of Pediatrics

Corresponding author:
Sarina K. Butzer
Kerpener Straße 62
50937 Cologne
Germany
E-Mail: sarina.butzer@uk-koeln.de

Supplementary Material

*Table S1: Detailed overview of the underlying oncological diagnoses of the 116 pediatric patients included in the study.*

| | Patients (n=116) | Episodes prior AMS intervention (n=270) | Episodes post AMS intervention (n=162) | Total number of Episodes (n=432) |
|---|---|---|---|---|
| **Oncological disease (%)** | | | | |
| ALL | 32 (27.6) | 71 (26.3) | 57 (35.2) | 128 (29.6) |
| AML | 13 (11.2) | 33 (12.2) | 22 (13.6) | 55 (12.7) |
| Mixed phenotype acute leukemia | 1 (0.9) | 0 | 5 (3.1) | 5 (1.2) |
| Hodgkin lymphoma | 7 (6.0) | 6 (2.2) | 4 (2.5) | 10 (2.3) |
| Non-Hodgkin lymphoma | 15 (12.9) | 42 (15.6) | 13 (8.0) | 55 (12.7) |
| Osteosarcoma | 3 (2.6) | 4 (1.5) | 4 (2.5) | 8 (1.9) |
| Ewing sarcoma | 7 (6.0) | 27 (10.0) | 14 (8.6) | 41 (9.5) |
| Neuroblastoma | 20 (17.2) | 42 (15.6) | 24 (14.8) | 66 (15.3) |
| Rhabdomyosarcoma | 5 (4.3) | 4 (1.5) | 11 (6.8) | 15 (3.5) |
| Nephroblastoma | 3 (2.6) | 5 (1.9) | 0 | 5 (1.2) |
| Clear cell sarcoma of the kidney | 1 (0.9) | 13 (4.8) | 0 | 13 (3.0) |
| Yolk Sac Tumor | 1 (0.9) | 3 (1.1) | 0 | 3 (0.7) |
| Hepatocellular carcinoma (HCC) | 1 (0.9) | 0 | 1 (0.6) | 1 (0.2) |
| Undifferentiated sarcoma | 1 (0.9) | 0 | 3 (1.9) | 3 (0.7) |
| Medulloblastoma | 3 (2.6) | 14 (5.2) | 2 (1.2) | 16 (3.7) |
| Astrocytoma | 1 (0.9) | 4 (1.5) | 0 | 4 (0.9) |
| Ependymoma | 1 (0.9) | 0 | 2 (1.2) | 2 (0.5) |
| ETMR | 1 (0.9) | 2 (0.7) | 0 | 2 (0.5) |

ALL: acute lymphoblastic leukemia, AML: acute myeloid leukemia; ETMR: Embryonal tumor with multilayered rosettes
